# Risk factors of opioid-induced adverse reactions in elderly male outpatients of Korea Veterans Hospital

**DOI:** 10.1186/s12877-018-0990-1

**Published:** 2018-11-29

**Authors:** Ji Young Kim, Joo Hee Kim, Jeong Yee, Soo Jin Song, Hye Sun Gwak

**Affiliations:** 1 Graduate School of Converging Clinical & Public Health, Ewha Womans University, Seoul, 03760 South Korea; 2 Department of Pharmacy, Korea Veterans Hospital, Seoul, 05368 South Korea; 3 College of Pharmacy & Division of Life and Pharmaceutical Sciences, Ewha Womans University, 52 Ewhayeodae-gil Seodaemun-gu, Seoul, 03760 Republic of Korea; 4 College of Pharmacy & Institute of Pharmaceutical Science and Technology, Ajou University, Suwon-si, 16499 South Korea

**Keywords:** Opioid, Adverse drug reactions, Male elderly patients

## Abstract

**Background:**

Risk factors associated with opioid-induced adverse reactions (OIARs) in the elderly population have not been well defined. The objective of this study was to determine effects of various risk factors on incidence of OIARs in male elderly patients.

**Methods:**

A retrospective cohort study in Korea Veterans Hospital was performed. Data were analyzed in male patients aged 65 years and older who received morphine, oxycodone, or codeine. Binomial variables describing patient-related and drug-related characteristics were constructed. Associations between these variables and frequency of OIARs were determined. Odds ratio (OR) and adjusted odds ratio (AOR) were calculated from univariate and multivariable analyses, respectively. Attributable risk was obtained by (1–1/OR)*100%.

**Results:**

Of 316 patients, 28% experienced at least one adverse event. The most common adverse events were gastrointestinal problems (*n* = 59) and central nerve system adverse effects (*n* = 20). The odds of OIARs in patients with opioid use ≥12 weeks was increased by 80% compared to those with opioid use < 12 weeks. Attributable risk of GABA analogues was 64~78% in constructed Models. Compared to codeine users, patients using morphine and oxycodone had 653 and 473% increased odds for OIARs, respectively. MME ≥ 60 mg/day had a 317% increased odds for OIARs (95% CI: 1.92–9.04) compared to MME < 60 mg/day. Opioid combination therapy had a 139% increased odds for OIARs compared to monotherapy.

**Conclusions:**

These findings have significant implications for clinical use of opioid in elderly patients. Our study suggests that low dose short-term use will pose less risk of OIARs for the elderly, whereas concomitant use of GABA analogues, strong opioids and dual-opioid therapy may increase the risk of OIARs. Therefore, clinician should carefully monitor patients when starting opioid therapy in older population.

## Background

Over the past 20 years, therapeutic use of opioids for both cancer and non-cancer pain has increased dramatically in developed countries [1, 2]. In many countries, opioids are now being introduced at earlier stages and used in higher doses in palliative care [3]. Global opioid consumption levels have also increased, especially in high income countries and regions including North America, Australia, New Zealand, and several western European countries. Consumption of opioids in these countries accounts for more than 90% of global use [4]. Starting from 5 mg morphine per capita use in 1980, global opioid consumption has increased to around 25 mg in 2015 [5]. Opioid use in the Republic of Korea has increased almost 7-fold between 1980 and 2015. Its consumption levels have exceeded levels reported in Japan.

In parallel with increased opioid dosages and patient access to opioids, there is an increasing concern about their adverse effects among health care providers, in addition to misuse and addiction problems. Opioid-induced adverse reactions (OIARs) are well-known. They may not differ between younger adults and older adults. However, some OIARs stemming from drug interactions are more specific to older adults considering their high incidence of polypharmacy [6]. More pronounced effects may also be seen in the elderly who are taking opioid drugs. For example, elderly patients are more likely to experience cognitive impairment and fall injuries when they are exposed to opioids and other centrally acting drugs. Although adverse events such as nausea, constipation, and bladder dysfunction are not life-threatening, they are more bothersome in older adults. They can lead to prompt discontinuation of the drug [7].

The extent and nature of OIARs depend on many factors. Previous studies have assessed the association between patient-factors (age, sex, and race) and incidence of some OIARs with short-term use in adults [8]. However, evidence regarding safety of opioid therapy in the elderly population is insufficient, and the studies that guide specific recommendations on opioid therapy in this age group are sparse. There are also the findings in the literature suggesting that chronic pain in the elderly population is often undertreated because of fear of OIARs [9]. Better understanding of risk factors associated with OIARs among the elderly could provide safer and more effective pain management.

Therefore, the objective of this cohort study was to focus on the elderly population and investigate patient-related and drug-related risk factors for OIARs with short-term and long-term use. The association between respective risk factors and commonly encountered OIARs such as gastrointestinal (GI) and central nerve system (CNS) adverse reactions was also analyzed.

## Methods

### Study population

This study is a retrospective study which was conducted at Korea Veterans Hospital, a 1400-bed hospital in Seoul, between January 2012 and December 2012. In this hospital, people ≥65 years of age comprise the majority (about 70% of over 4000 patients per year) of the patients and opioid initiations are common among veterans with chronic pain. All ambulatory patients with the following characteristics were included: 1) was administered with a single component oral opioid (codeine phosphate, morphine sulfate, or oxycodone hydrochloride) for at least a day between January 1, 2012 and December 31, 2012, 2) were at least 65 years of age when their first opioid was prescribed. In our study period, opioids were prescribed for several clinical symptoms such as cancer pain as well as non-cancer pain including various types of neuropathic pain. The departments from which opioids were prescribed were as follow: pain management clinic, internal medicine, family medicine, respiratory medicine, thoracic surgery, orthopedic surgery, otorhinolaryngology, hematology and oncology. From patients whose data were complete for analysis, we excluded female patients because the sample proportion for females was insufficient to analyze gender interactions on the effects of interest [10]. Patient whose opioids were frequently switched were also excluded since it is ambiguous as to which opioid group such patients should be placed in. This study was approved by the Institutional Review Board (IRB # BOHUN 2017–10-005).

### Data collection

Baseline patient characteristics collected from electronic medical records included age, weight, height, body mass index (BMI), alcohol consumption, smoking, underlying diseases, indication of opioid use, opioid type and maintenance doses, duration of opioid therapy, previous experience of opioid use, concomitant drug use, opioid combination therapy, and OIARs.

Only the opioid use from the first visit during our study period (year 2012) was included. Cumulative dose (expressed in MME (morphine milligram equivalents)) was calculated by using conversion factors of 0.15 for codeine and 1.5 for oxycodone. Duration of opioid therapy (defined as continuous use of opioids with a gap of no greater than 30 days) was divided into two groups based on 12 weeks according to Langzeitanwendung von Opioiden bei nicht tumorbedingten Schmerzen (LONTS) Guideline.

Previous experience of opioid use was defined as opioid use in the year before their first opioid prescription in 2012. Concomitant drugs during opioid use included tramadol, fentanyl patch, benzodiazepines (alprazolam, chlordiazepoxide, clobazam, clonazepam, diazepam, flurazepam, lorazepam, midazolam, and triazolam), amitriptyline, and gamma-aminobutyric acid (GABA) analogues (gabapentin and pregabalin).

### Outcome measures

The primary outcome was to estimate the OIARs in association with baseline and clinical variables. OIAR was defined as the presence of one or more events among GI problems (e.g. constipation, nausea, vomiting and abdominal pain), dizziness, drowsiness, urinary retention, skin rash, respiratory depression, and other symptoms within 4 weeks of opioid prescription. Secondary outcomes were consisted of GI (GI problems) and CNS (dizziness and drowsiness) OIARs.

### Statistical analysis

Chi-squared test or Fisher’s exact test was used to compare categorical variables between patients with OIARs and those without OIARs and unpaired t test were used for continuous variables. Multivariable logistic regression analysis was used to identify independent risk factors for OIARs. The reference group for opioid type in statistical models was defined as codeine, which is less potent than morphine and oxycodone. Factors having *p*-values < 0.05 from univariate analysis along with a strong confounder of age (if necessary) were included in multivariable analysis and listwise deletion was used for missing values. Variance inflation factor (VIF) was calculated to evaluate multicollinearity using “Allison’s criteria (VIF>2.5)” [11]. Attributable risk was calculated by (1–1/OR)*100%. *P* values less than 0.05 were considered statistically significant. All statistical analyses were carried out using the Statistical Package for Social Sciences (SPSS), version 20.0 for Windows (SPSS Inc., Chicago, IL, USA).

## Results

A total of 332 patients were prescribed in the outpatient clinic during the study period. Twelve patients were excluded, including three female subjects (0.9%) and nine subjects (2.7%) with frequent switch of opioids. Accordingly, data from 316 patients who took opioids were used for final analysis. Indications of opioid use were cancer pain (*n* = 81), dyspnea (*n* = 61), back pain (*n* = 52), neuropathy (*n* = 52), post-surgery pain (*n* = 6), traumatic pain (*n* = 5), and other pains (*n* = 59, including leg pain, intermittent claudication, shoulder pain, knee pain, and chest pain). OIARs included GI problems (*n* = 59), dizziness and drowsiness (*n* = 20), urinary retention (*n* = 7), skin rash (*n* = 5), respiratory depression (*n* = 2), and others (*n* = 31, including sweating, blurred vision, appetite loss, headache, and accommodation disturbance).

As shown in Table 1, the frequency of OIARs was 28.2%. Patients’ mean age was 70.9 ± 5.1 years. A total of 131 (41.5%) patients were ≥ 70 years of age. More than 40% of patients were obese (BMI ≥ 25 kg/m^2^). Frequencies of alcohol intake and smoking were 95 (32.3%) and 72 (24.2%), respectively. Drugs concurrently administered with opioids included tramadol or fentanyl patch (*n* = 143), benzodiazepines (*n* = 92), amitriptyline (*n* = 87), and GABA analogues (*n* = 160). Various opioids were used, including oxycodone (*n* = 111), codeine (*n* = 94), and morphine (*n* = 78).

**Table 1:** Demographic characteristics of the study population

Characteristics	No (%)	Adverse reaction No (%)		P
		Presence (*n* = 89)	Absence (*n* = 227)	
Age (years)				
< 70	185 (58.5)	52 (58.4)	133 (58.6)	0.979
≥ 70	131 (41.5)	37 (41.6)	94 (41.4)	
Mean ± S.D.	70.8 ± 5.6	70.3 ± 5.0	71.0 ± 5.8	0.327
Weight (kg)^a^				
< 65	141 (49.5)	40 (47.1)	101 (50.5)	0.595
≥ 65	144 (50.5)	45 (52.9)	99 (49.5)	
Mean ± S.D.	65.8 ± 10.8	66.5 ± 11.2	65.5 ± 10.6	0.467
Height (cm)^a^				
< 165	138 (48.4)	40 (47.1)	98 (49.0)	0.764
≥ 165	147 (51.6)	45 (52.9)	102 (51.0)	
Mean ± S.D.	164.9 ± 5.5	165.4 ± 5.3	164.7 ± 5.6	0.351
BMI (kg/m^2^)^a^				
< 25	169 (59.3)	53 (62.4)	116 (58.0)	0.494
≥ 25	116 (40.7)	32 (37.6)	84 (42.0)	
Mean ± S.D.	24.2 ± 3.6	24.3 ± 3.8	24.1 ± 3.5	0.690
Alcohol^b^				0.498
Yes	95 (32.3)	25 (29.4)	70 (33.5)	
No	199 (67.7)	60 (70.6)	139 (66.5)	
Smoking^c^				0.181
Yes	72 (24.2)	25 (29.4)	47 (22.1)	
No	226 (75.8)	60 (70.6)	166 (77.9)	
Cardiovascular disease				0.369
Yes	245 (77.5)	72 (80.9)	173 (76.2)	
No	71 (22.5)	17 (19.1)	54 (23.8)	
Diabetes mellitus				0.036
Yes	127 (40.2)	44 (49.4)	83 (36.6)	
No	189 (59.8)	45 (50.6)	144 (63.4)	
Hepatic disease				0.205
Yes	38 (12.0)	14 (15.7)	24 (10.6)	
No	278 (88.0)	75 (84.3)	203 (89.4)	
Renal disease				0.814
Yes	30 (9.5)	9 (10.1)	21 (9.3)	
No	286 (90.5)	80 (89.9)	206 (90.7)	
Duration of opioid use				< 0.001
< 12 weeks	195 (61.7)	42 (47.2)	153 (67.4)	
≥ 12 weeks	121 (38.3)	47 (52.8)	74 (32.6)	
Previous opioid use				0.088
Yes	193 (61.1)	61 (68.5)	132 (58.1)	
No	123 (38.9)	28 (31.5)	95 (41.9)	
Opioid combination				0.020
Yes	33 (10.4)	15 (16.9)	18 (7.9)	
No	283 (89.6)	74 (83.1)	209 (92.1)	
Tramadol or fentanyl^*^				0.494
Yes	143 (45.3)	43 (48.3)	100 (44.1)	
No	173 (54.7)	46 (51.7)	127 (55.9)	
Benzodiazepine				0.012
Yes	92 (29.1)	35 (39.3)	57 (25.1)	
No	224 (70.9)	54 (60.7)	170 (74.9)	
Amitriptyline				0.208
Yes	87 (27.5)	29 (32.6)	58 (25.6)	
No	229 (72.5)	60 (67.4)	169 (74.4)	
GABA analogue				< 0.001
Yes	160 (50.6)	67 (75.3)	93 (41.0)	
No	156 (49.4)	22 (24.7)	134 (59.0)	
Opioid type^d^				< 0.001
Codeine	94 (33.2)	5 (6.8)	89 (42.6)	
Oxycodone	111 (39.2)	35 (47.3)	76 (36.4)	
Morphine	78 (27.6)	34 (45.9)	44 (21.1)	
MME				
< 60	107 (33.9)	9 (10.1)	98 (43.2)	< 0.001
≥ 60	209 (66.1)	80 (89.9)	129 (56.8)	
Mean ± S.D.	98.7 ± 145.8	131.1 ± 104.2	85.9 ± 157.5	0.013

^*^fentanyl patch. *BMI* body mass index, *MME* morphine milligram equivalent

^a^31 missing data for weight, height, and BMI

^b^22 missing data for alcohol consumption

^c^18 missing data for smoking

^d^Monotherapy only

Presence of diabetes mellitus (DM), duration of opioid therapy, benzodiazepine use, GABA analogue use, opioid type, opioid combination therapy, and MME were significant factors of OIARs in univariate analysis (Tables 1 and 2). To analyze the respective effect of single opioid type and combination therapy on adverse reactions, two separate models were constructed for multivariable analysis. Model I included age, DM, duration, benzodiazepine, GABA analogue, and opioid type, while Model II included combination therapy instead of opioid type. Since there was multicollinearity between opioid type and MME, Model III was additionally constructed by including MME instead of opioid type of Model I. VIF values for morphine, oxycodone, and MME ≥ 60 were 6.18, 6.38, and 6.00, respectively.

**Table 2:** Univariate and multivariable regression analyses to identify predictors of opioid-induced adverse reactions

Characteristics	Unadjusted OR (95% CI)	Model I		Model II		Model III	
		Adjusted OR (95% CI)	Attributable risk	Adjusted OR (95% CI)	Attributable risk	Adjusted OR (95% CI)	Attributable risk
Age ≥ 70	1.01 (0.61–1.66)						
Diabetes mellitus	1.70 (1.03–2.78)^*^						
Duration ≥12 weeks	2.31 (1.40–3.82)^**^	1.79 (0.99–3.24)		1.81 (1.05–3.14)^*^	44.9	1.80 (1.05–3.08)^*^	44.3
Benzodiazepine	1.93 (1.15–3.25)^*^						
GABA analogue	4.39 (2.53–7.60)^***^	2.80 (1.42–5.54)^**^	64.3	4.55 (2.57–8.05)^***^	78.0	2.98 (1.66–5.33)^***^	66.4
Oxycodone^a^	8.20 (3.06–21.97)^***^	5.73 (2.08–15.77)^***^	82.6				
Morphine^a^	13.75 (5.03–37.61)^***^	7.53 (2.60–21.86)^***^	86.7				
Combination therapy	2.35 (1.13–4.91)^*^			2.39 (1.03–5.53)^*^	58.1		
MME ≥ 60	6.75 (3.23–14.12)^***^					4.17 (1.92–9.04)^***^	76.0

For model I, age, diabetes, duration, benzodiazepine, GABA analogue, and opioid type were included for analysis. For model II, age, diabetes, duration, benzodiazepine, GABA analogue, and opioid combination therapy were included for analysis. For model III, age, diabetes, duration, benzodiazepine, GABA analogue, and morphine equivalent dose were included for analysis

^a^Compared to codeine. *MME* morphine milligram equivalent

^*^P < 0.05, ^**^P < 0.01, ^***^P < 0.001

Multivariable analysis showed that duration of opioid use, concomitant use of GABA analogue, opioid type, opioid combination therapy, and MME were significant factors of OIARs after controlling variables with *P* value of less than 0.05. Patients with opioid use ≥12 weeks had a 79% increased odds of OIAR compared to those with opioid use < 12 weeks (OR = 1.79). Attributable risk of GABA analogues was 64~78% in constructed Models. Compared to codeine (the least potent) users, patients using morphine and oxycodone had 653 and 473% increased odds of OIAR, respectively. MME ≥ 60 mg/day showed a 317% increased odds (OR = 4.17, 95% CI: 1.92–9.04) of OIAR compared to MME < 60 mg/day. Opioid combination therapy had a 139% increased odds of OIAR compared to monotherapy.

In analysis of GI AR (Table 3), similar results to those described above were obtained. Duration of opioid therapy ≥12 weeks (AOR: 2.00), GABA analogue (AOR: 2.67–3.92), morphine (AOR: 5.31, 95% CI: 1.60–17.59) or oxycodone use (AOR: 3.75, 95% CI: 1.20–11.72), and MME (AOR: 3.38, 95% CI: 1.34–8.52) were significant factors of GI and urinary AR after adjusting for confounders.

**Table 3:** Univariate and multivariable regression analyses to identify predictors of opioid-induced gastrointestinal adverse reactions

Characteristics	Unadjusted OR (95% CI)	Model I		Model II		Model III	
		Adjusted OR (95% CI)	Attributable risk	Adjusted OR (95% CI)	Attributable risk	Adjusted OR (95% CI)	Attributable risk
Age ≥ 70	1.00 (0.56–1.78)						
Diabetes mellitus	1.78 (1.00–3.16)^*^						
Duration ≥12 weeks	2.55 (1.43–4.55)^**^	2.00 (1.03–3.88)^*^	49.9	1.98 (1.06–3.71)^*^	49.5	2.03 (1.10–3.72)^*^	50.6
GABA analogue	3.85 (2.01–7.37)^***^	2.14 (0.97–4.75)		3.92 (2.00–7.67)^***^	74.5	2.67 (1.35–5.30)^**^	62.5
Oxycodone^a^	5.25 (1.73–15.91)^**^	3.75 (1.20–11.72)^*^	73.3				
Morphine^a^	8.84 (2.89–26.99)^***^	5.31 (1.60–17.59)^**^	81.2				
Combination therapy	2.51 (1.14–5.52)^*^			2.33 (0.96–5.64)			
MME ≥ 60	5.58 (2.31–13.46)^***^					3.38 (1.34–8.52)^*^	70.4

For model I, age, diabetes, duration, GABA analogue, and opioid type were included for analysis. For model II, age, diabetes, duration, GABA analogue, and opioid combination therapy were included for analysis. For model III, age, diabetes, duration, GABA analogue, and morphine equivalent dose were included for analysis

^a^Compared to codeine. *MME* morphine milligram equivalent

^*^P < 0.05, ^**^P < 0.01, ^***^P < 0.001

In the case of CNS AR (Table 4), GABA analogue was the only significant factor in multivariable analysis. The attributable risk of concomitant use of GABA analogues was 85~90%.

**Table 4:** Univariate and multivariable regression analyses to identify predictors of opioid-induced central verve system adverse reactions

Characteristics	Unadjusted OR (95% CI)	Model I		Model II	
		Adjusted OR (95% CI)	Attributable risk	Adjusted OR (95% CI)	Attributable risk
GABA analogue	9.76 (2.23–42.82)^**^	9.81 (2.23–43.09)^**^	89.8	6.53 (1.45–29.43)^*^	84.7
Morphine^a^	2.79 (1.12–6.97)^*^				
MME ≥ 60	10.60 (1.40–80.30)^*^			5.96 (0.76–46.97)	

For model I, GABA analogue, and morphine were included for analysis. For model II, GABA analogue, and morphine equivalent dose were included for analysis. *MME* morphine milligram equivalent

^a^Compared to codeine and oxycodone

^*^P < 0.05, ^**^P < 0.01

## Discussion

Both short-term and long-term use of opioids is commonly associated with multiple adverse effects [12]. Across studies, up to 80% of patients experience at least one adverse event. The most common adverse events are GI and CNS adverse effects [13]. In our study population, approximately 28% of patients had at least one adverse effect. The most common adverse effects were GI and CNS problems.

Opioid-induced bowel dysfunction (OIBD) is a well-known and frequent event, with estimated prevalence of 15 to 90% based on a recent systematic review [14]. Our study revealed that 59 (19%) of 316 patients reported GI problems. This prevalence was relatively low. This wide variation in frequency of OIBD can be attributed to heterogeneity of study design and population, including differences in age, gender, underlying pathology, perception of constipation, and definition of constipation. Particularly, a higher rate of constipation (90%) was reported in the study including transdermal fentanyl and sustained release oral morphine compared to the one including ‘weak’ opioids such as codeine. One third (33%) of our study patients were codeine users.

One of the main findings of our study was that concomitant use of GABA analogue such as pregabalin and gabapentin increased the odds of OIARs by 180~355%. It increased the odds of CNS adverse events by 553~881%. This result suggests that close monitoring for potential risks of OIARs with co-prescribing GABA analogues is warranted. Until recently, gabapentin and pregabalin have been considered to have a low profile of drug interactions. They have been used as adjunctive drugs to opioids for clinical management of neuropathic and chronic pain [15]. Notably, after analyzing the safety in synthesized randomized trials, it has been revealed that a combination of opioid and gabapentin can cause more frequent side effect-related trial dropouts compared to gabapentin alone [16]. More and more gabapentin prescriptions are prescribed. Therefore, caution is needed.

More than half of our study patients received co-prescription of opioids with GABA analogues. In comparison, a combination of opioids with other CNS depressants such as benzodiazepines and amitriptyline was used in less than one third of our patients. This might be because their additive effects on sedation have been well recognized. The American Geriatrics Society (AGS) has been emphasizing adverse drug interactions between opioids and CNS active drugs including benzodiazepines and tricyclic antidepressants [17]. Reported data of opioid prescription-related deaths have also drawn increasing alarm. The use of benzodiazepine has been frequently cited as a contributing factor [18].

Potential harms and benefits of dual-opioid therapy are under increasing discussion. In our study, administration of opioid combination increased the odds of both all OIARs and GI AR by 130% compared to monotherapy. There are controversial issues in terms of efficacy and safety of morphine-oxycodone combination for both acute and chronic pain [19]. At the moment, information is still new. Therefore, adverse effects of opioid combination therapy should be closely monitored as data regarding dosing schedule or dose levels of optimal combination in elderly population are poorly available.

Although less pronounced, the frequency of all OIARs and GI adverse events in our patients was increased with long-term therapy (12 weeks and over). In addition, MME over 60 mg resulted in attributable risk of 76 and 70% in all OIARs and GI adverse events, respectively. Recent studies have also shown that higher opioid dose is associated with the risk of OIARs such as fractures, depression, opioid overdose, and misuse [20]. One study on adults aged over 60 years has found that patients taking opioids at doses of MME over 50 mg have a two-fold increase in fracture risk. More than one third of these subjects were associated with inpatient care [21]. There is a lack of evidence to prove that benefits will outweigh potential harm when opioids are given long-term, especially for high-risk group [22]. According to a new study, long-term use of opioids (12 weeks and over) is associated with worse functional status, including inability to work. Adverse events such as depression, opioid dependence, and overdose were also more common in those taking opioids long-term compared to those who had short-term use [23].

A couple of reviews have been performed to address whether one opioid is superior to another with respect to tolerability. It has been claimed there are no differences in the incidence of adverse effects between morphine and meperidine [24]. Other authors have found that, at equi-analgesic doses, patients who receive meperidine have lower incidence of nausea and vomiting compared to those who receive morphine [8]. Our study has found that strong opioids (morphine and oxycodone) are associated with higher risk of all OIARs and GI problems compared to codeine. Due to co-linearity between potency of opioid and opioid dose, patterns of association between these factors and the risk of OIARs have shown to be consistent in statistical models. Risks of OIARs in patients receiving strong opioids were high probably because they were taking high dose.

Our study has several limitations. Since our dataset solely came from adverse events detected by chart review, rates of OIARs appeared to be lower compared to those by incident reporting or controlled trials [25]. Moreover, elderly patients tend not to report symptoms of OIARs which often cannot be differentiated from age-related issues. Another limitation was that our study was limited to a population of patients eligible for Veterans benefit program. Most of them were male individuals with chronic illness. In our study, female patients were not included in analysis because the sex ratio of the study population is extremely uneven and sex differences in OIARs were reported in previous literature [26–29]. About 78 and 40% of our study population had cardiovascular disease and diabetes, respectively. Therefore, our findings may not be applicable to the general population of opioid users.

Nevertheless, our findings have important clinical implications. Due to pharmacokinetic and pharmacodynamic differences compared to younger patients, elderly patients may have an increased risk of OIARs. Also, older patients exhibit a higher likelihood of polypharmacy and drug-drug interactions with opioids.

## Conclusions

Our data suggest that concomitant use of GABA analogue, strong opioids, and dual-opioid therapy may increase the risk of OIARs in the elderly. Low dose short-term use showed less risk of OIARs in our study population. Therefore, it is recommended that opioids be prescribed at the lowest effective dose to minimize OIARs, and clinician should carefully monitor patients when starting opioid therapy in older population.

## Abbreviations

AOR: Adjusted odds ratio
BMI: Body mass index
CNS: Central nerve system
DM: Diabetes mellitus
GABA: Gamma-aminobutyric acid
GI: Gastrointestinal
MMEs: Morphine milligram equivalents
OIARs: Opioid-induced adverse reactions
OR: Odds ratio
